# Neuroorganoleptics: Organoleptic Testing Based on Psychophysiological Sensing

**DOI:** 10.3390/foods10091974

**Published:** 2021-08-24

**Authors:** João Valente, Leonor Godinho, Cristina Pintado, Cátia Baptista, Veronika Kozlova, Luís Marques, Ana Fred, Hugo Plácido da Silva

**Affiliations:** 1 Instituto Politécnico de Castelo Branco (IPCB), Av. Pedro Álvares Cabral 12, 6000-084 Castelo Branco, Portugal; veronika@ipcb.pt; 2 BrainAnswer, Lda., R. Eng. Pires Marques 61, 6000-406 Castelo Branco, Portugal; euluismarques@gmail.com; 3 Department of Bioengineering (DBE), Instituto Superior Técnico (IST), Av. Rovisco Pais 1, 1049-001 Lisboa, Portugal; leonor.godinho@tecnico.ulisboa.pt (L.G.); afred@lx.it.pt (A.F.); hsilva@lx.it.pt (H.P.d.S.); 4 CATAA—Associação Centro de Apoio Tecnológico Agro-Alimentar, Zona Industrial de Castelo Branco, Rua A, 6000-459 Castelo Branco, Portugal; cmiguel@cataa.pt (C.P.); catia.baptista@cataa-cei.pt (C.B.); 5 Instituto de Telecomunicações (IT), Av. Rovisco Pais 1, 1049-001 Lisboa, Portugal

**Keywords:** consumer behavior research, sensory analysis, organoleptics, psychophysiological data, neuroorganoleptics

## Abstract

There is an increasing interest, in consumer behaviour research related to food and beverage, in taking a step further from the traditional self-report questionnaires and organoleptic properties assessment. With the growing availability of psychophysiological data acquisition devices, and advancements in the study of the underlying signal sources seeking affective state assessment, the use of psychophysiological data analysis is a natural evolution in organoleptic testing. In this paper we propose a protocol for what can be defined as neuroorganoleptic analysis, a method that combines traditional approaches with psychophysiological data acquired during sensory testing. Our protocol was applied to a case study project named MobFood, where four samples of food were tested by a total of 83 participants, using preference and acceptance tasks, across three different sessions. Best practices and lessons learned regarding the laboratory setting and the acquisition of psychophysiological data were derived from this case study, which are herein described. Preliminary results show that certain Heart Rate Variability (HRV) features have a strong correlation with the preferences self-reported by the participants.

## 1. Introduction

In recent years, increasing attention has been given to the challenge of understanding the reasons behind consumers’ choices and selections, particularly in the food and beverage industry [1]. One of the big questions researchers are trying to answer nowadays is whether psychophysiological responses can provide new insights into the decision-making process of consumers. Specifically concerning edible products, there is a great interest in obtaining accurate and objective data about the sensory perception of food and beverages and, consequently, about consumption intention [2,3,4]. This field of study can be greatly reinforced through the development of new and more in-depth protocols of sensory analysis, capable of complementing self-reporting questionnaires with the use of psychophysiological traits.

When it comes to tasting food, the authors of [5] defined three main emotional processing levels: The low processing level, when subjects integrate information from the five senses through psychophysiological, unconscious, processes; the intermediate processing level, when subjects connect their perceptions to past experiences and information already present in their memory, through both conscious and unconscious processes; and lastly, the high processing level, when subjects consciously recognise what the samples they are tasting are able to express which emotions they evoke (e.g., if the taste matched their expectations, if the visual appearance of the product is pleasant, among many others) [6].

In addition to organoleptic assessment, the traditional sensory testing methods rely on self-report questionnaires, as they present unquestionable advantages, such as being easy to deploy and being cost-effective [7,8]. However, this approach also presents some limitations. The main one is that, by only being able to target the high processing level and obtaining conscious responses, they are not depicting the low and intermediate processing levels, potentially leaving out relevant unconscious processes present in these [9]. Moreover, self-report questionnaires, by targeting the high processing level, are also subjected to the inherent difficulty of subjects having to reconstruct and interpret their thoughts and motivations, and/or to verbally express and assess them [10,11]. This, combined with the fact that the expression and assessment of emotions (e.g., liking, pleasure, etc.) varies across cultures and languages [5,11], can make the results of self-assessment questionnaires only representative by enrolling a sufficiently high number of subjects. Finally, the last constraint of self-assessment questionnaires is that they only obtain declared opinions, not revealing responses that the consumers may not wish to disclose (e.g., because they are socially undesirable) [10,11,12].

The use of psychophysiological data has the potential to overcome these limitations, with the advantage of being able to depict the low and intermediate processing levels, and not depending on the verbal declared expression of subjects to obtain measurable results [9,13]. The physiological data and derived features used in this area can be divided into two main groups, one related to recording the activity of the brain, by electroencephalography (EEG), and another using peripheral measurements. The latter is generally easier to deploy, and includes signals such as ElectroDermal Activity (EDA), Heart Rate (HR), Skin Temperature (ST), Blood Pressure (BP), pupillometry, eye-tracking (ET), and facial expressions [5,9,12]. These signals are common in studies related to neuropsychology and affective state assessment, since they provide valuable insights into the Autonomic Nervous System (ANS) [14].

The ANS integrates the Sympathetic Nervous System (SNS), which conducts the body’s responses in case of a threat, and the Parasympathetic Nervous System (PNS), which is in charge of homeostasis [9,15]. Both the SNS and PNS control the cardiac dynamics (e.g., HR), the dilation and constriction of blood vessels’ diameters (BP), pupils (pupillometry and ET), bronchial tubes (respiration), and the contraction and relaxation of muscles, modulating these responses according to the environment and stimuli [16]. The EDA response is, on the other hand, only influenced by the SNS, being more associated with stressful or arousing events [17]. As a consequence of many of these responses (e.g., muscle constriction/relaxation, sweating and vasoconstriction/vasodilatation), the body temperature varies, which makes it an indirect measure of ANS responses [16]. All in all, the ANS is responsible for the unconscious responses of the human body, which are associated with an emotional response, arousal, and stress [14].

That being said, using psychophysiological sensing in food and beverage sensory testing is a natural complement to traditional consumer research methods. This approach can be designated as neuroorganoleptics, and it allows reaching every processing level involved in the tasting of food and beverages, enabling researchers and other stakeholders to more accurately obtain information about the consumer’s food perceptions and preferences. It has only recently started to be investigated in the state-of-the-art, as shown by a group of researchers from the University of Melbourne, who applied this novel sensory analysis approach to assess consumers’ acceptability of different samples of beer [18] and chocolate [19].

With this in mind, this paper describes a comprehensive protocol for neuroorganoleptic testing. It has been applied to a case study project (MobFood), further described in Section 3. The case study had the goal of analysing the acceptability and consumption intention of four different food samples that were part of a breakfast kit, combining the typical self-reporting questionnaires with psychophysiological data analysis. The signals included in this protocol were EDA, Photoplethysmography (PPG), Respiration (PZT), Electrocardiography (ECG), and Eye-Tracking (ET). A webcam and microphone were also used to record the environmental context of the experiment.

The remainder of the work is organised as follows: Section 2 describes the design considerations of the proposed neuroorganoleptic testing protocol; Section 3 details the application of the devised protocol to the MobFood case study; Section 4 summarises the main findings and results from this case study; and, finally, Section 5 provides a brief discussion and future work guidelines.

## 2. Neuroorganoleptic Testing Protocol

### 2.1. Assessment Scales

This protocol expands standardised tests with sensory analysis tasks, more specifically, affective tests designed to assess acceptance, liking, or product preference. These methods do not require experienced assessors, and include two main types of quantitative tests: preference and acceptance [20]. Both types are present in our protocol, and questions to assess the consumers’ buying intentions are also included. At the beginning of the protocol, the tasks consist of presenting digital visual stimuli (images of the samples in the computer) for preference tests. After that, the physical samples are used for the remaining acceptance and preference tests.

In preference tests, the assessors are given two or more samples and required to indicate which one they prefer [21]. If more than two samples are presented, the assessors rank them by their preference [22]. This is a simple test to implement and also easy to analyse statistically, since the values of significance between rank sums are defined for the number of samples compared and the number of assessors [23]. A disadvantage of this test is that the degree of satisfaction is not determined.

On the other hand, in acceptance tests, assessors are presented with the samples and are required to assess the degree of liking of the product, globally, or of a certain attribute on a scale [22]. The most widely used scale is the 9-point hedonic scale, which is a balanced bipolar scale with a neutral point at the center, having four negative and four positive options on each side [24]. In this protocol, the scale is horizontal and has a label on the left extreme, center, and right extreme (e.g., ‘Extremely unpleasant’, ‘Indifferent’ and ‘Extremely pleasant’). This scale has the advantage of being accessible and easy to use for both participants and researchers, due to its categorical nature and limited set of options [25]. Statistically, analysis of variance (if normality of data is proved) and mean values comparison tests (e.g., Duncan’s multiple range test) are usually conducted to analyse the results of food and beverage tests using scales [26].

Another type of input proposed in our protocol is the Net Promoter Score (NPS), a 10-point scale used for questions asking if the consumer would recommend the product [27]. Depending on the answer, the assessor is grouped into one of the categories: promoter (9–10 points); passively satisfied (7–8 points); and detractor (0–6 points).

Finally, at the end of the protocol there are questions presented with the purpose of characterising the level of food neophobia of the subjects (Table A1). These questions use the 7-point Likert scale, in order to assess the attitude of the subjects towards the statements presented [28]. The extremes of this scale are labelled with ‘Strongly disagree’ and ‘Strongly agree’.

### 2.2. Laboratory Setting

Regarding the laboratory setting, the international standard of sensory analysis guidelines regarding staff [29], methodology and test room design [30] are taken into account as the primary reference.

**Figure 1:**
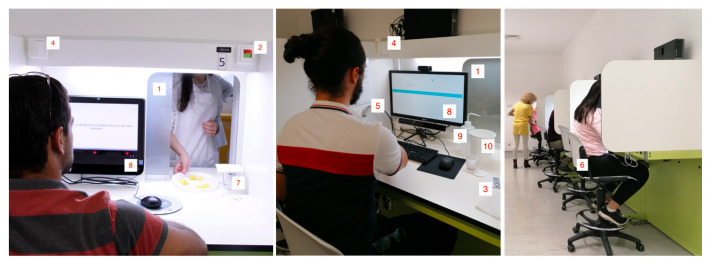
Overall layout of the individual test booths and room for neuroorganoleptic assessment.

The experimental protocol is carried out in individual testing booths, in order to avoid distractions and assessors influencing the answers of other peers through gestures or expressions. These individual booths are placed on the opposite side of the wall of the sample preparation area, and each booth possesses a horizontal sliding door (1 in Figure 1) that is used to provide the samples to the assessors. The door is kept closed in the remaining moments, to prevent the assessors from watching the sample preparation.

An important consideration taken into account in this protocol is to reduce the external interference to the bare minimum. To minimise ambient noise, one of the precautions is for the assessors to signal they are ready to receive the samples through a light signaling system connecting the booth to the preparation area. The signal switch (2 in Figure 1) is placed over the sliding door and, as the assessors flip the switch, a light is activated on the sample preparation area. Moreover, as the protocol involves the assessors asking for a particular sample at a particular time, a selection system using paper cards (three in Figure 1) is proposed. The assessors have a set of cards, each with a type of sample written on it, and these are used to request the samples to the operators in the preparation area, as a form of eliminating potential language barriers and avoiding talking (which would be an interference to the remaining assessors).

Besides noise, the temperature is kept at a level considered by the assessors to be comfortable. In our experimental setting, we seek to maintain a neutral environment, ensuring that the space is free from odors, and that the walls and surfaces are white, as well as the lighting option (four in Figure 1) used throughout the whole experiment.

Furthermore, each booth is numbered with a tag above the sliding door, and has a small sink and a water faucet (5 in Figure 1). The assessors are seated in an adjustable revolving stool with back support (six in Figure 1) and, on the table in front of them, there is a glass covered by a napkin (seven in Figure 1), the aforementioned paper cards and finally, the computer (eight in Figure 1) used to answer the questions present in the protocol. Lastly, as this experiment involved physiological data acquisition, on top of the table there is also a bottle of hydrogen peroxide (nine in Figure 1) and cotton, to be used during the placement of the sensors’ electrodes (detailed in Section 2.3.3). A small trash can (10 in Figure 1) can be used by the participant to dispose of electrodes and other used items.

As the experiment in our case study took place during the *COVID-19* pandemic, special care was taken, such as having an empty booth between two occupied booths to ensure a 2 m distance between assessors (as seen on the right of Figure 1). The assessors were, however, asked to remove their masks during the protocol.

### 2.3. Collected Data

#### 2.3.1. Self-Reporting Questionnaire

**Figure A1:**
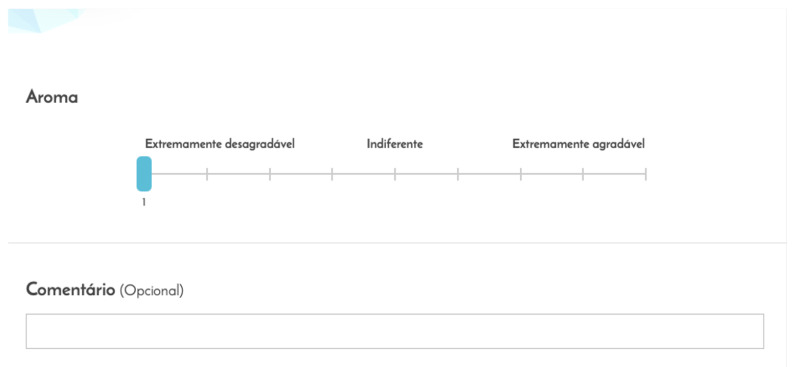
Example of acceptance question of the protocol using an 9-point hedonic scale concerning aroma. Translation from top to bottom: ‘Aroma’, ‘Extremely unpleasant’, ‘Indifferent’, ‘Extremely pleasant’, ‘Comments (optional)’.

The questionnaire was presented and filled in using the *BrainAnswer* platform (http://brainanswer.pt, accessed on 23 August 2021). Each question was presented individually, occupying the whole screen, in order to focus the attention of the assessor (example in Figure A1). The assessors used the keyboard to answer written questions and the mouse to select points in a scale, choose the preferred stimulus, or order stimuli. All of the questions were written in clear, simple terms, in the native language of the assessors.

#### 2.3.2. Eye-Tracking, Webcam and Audio

Regarding eye-tracking, our protocol used a GP3 Eye Tracker from Gazepoint (http://apps.usd.edu/coglab/schieber/eyetracking/Gazepoint/pdf/GazepointSpecs.pdf, accessed on 23 August 2021), a portable camera for which the main specifications are presented in Table 1. Moreover, throughout the whole experiment, the assessors were recorded both with the computer webcam and microphone. These sources are important, on one hand, for the validation of the experiment, as it allows the researchers to verify in post-processing if external influencing events have occurred when a signal is very abnormal (e.g., someone walked to the booth or ambient noise). On the other hand, the webcam also allows the analysis of facial expressions.

#### 2.3.3. Physiological Data

During the experiment, various psychophysiological signals were collected, which is a significant difference from standard protocols. All of these acquired signals allow the ANS response study, by analysing the changes over time on the Heart Rate Variability (HRV) [31], respiration levels, and skin conductance. In our case, data were acquired using the *BITalino (r)evolution Plugged BT* system [32,33], a Bluetooth wireless acquisition unit. This device is used in a 10-bit resolution and 1 kHz sampling frequency configuration; Table 2 describes the main technical specifications of the acquisition unit.

This system integrates the sensors selected for assessing the neurosensory component, characterised as follows. It is important to highlight that in the equations, $V_{CC}=3.3$ V (the operating voltage of the circuit), ADC is the value sampled from the channel (digital code assigned by the analog-to-digital converter to the input voltage), and *n* is the number of bits of the channel.

**Figure 2:**
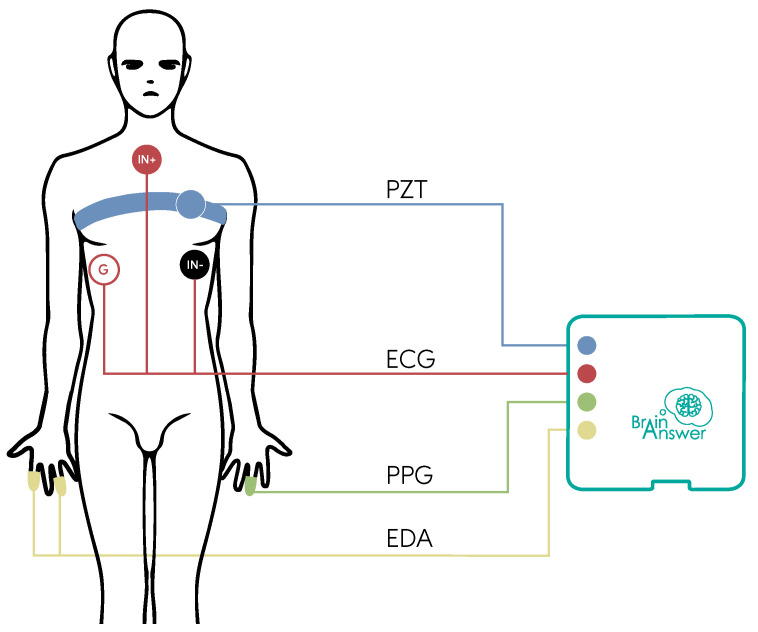
Schematic of the placement for the ECG (red) electrodes, and PZT (blue), PPG (green), and EDA (yellow) sensors.

The electrocardiography (https://bitalino.com/storage/uploads/media/revolution-ecg-sensor-datasheet-revb-1.pdf, accessed on 23 August 2021) (ECG) sensor uses three pre-gelled disposable Ag/AgCl *Eurotrode* electrodes (https://app.brainanswer.pt/store/#!/ECG-Electrodes-51mm-100pcs/p/205640017/category=52356463, accessed on 23 August 2021). Each electrode has an associated color, where the white electrode (ground) is placed on the right thoracic wall, the black electrode (IN-) is placed on the left thoracic wall (V5 precordial lead located in the left anterior axillary line), and the red electrode (IN+) is placed below the neck, on the manubrium (see Figure 2). The ECG sensor characteristics are described in Table 3, and its transfer function is given by Equation (1), in which the gain *G* is 570 (customised for this application to maximise the signal-to-noise ratio).

(1) $$ECG\left(V\right)=\frac{\left(\frac{ADC}{2^{n}}-\frac{1}{2}\right)\times V_{CC}}{G_{ECG}}$$

The piezoelectric respiration (https://bitalino.com/storage/uploads/media/pzt-sensor-datasheet-revb.pdf, accessed on 23 August 2021) (PZT) sensor used is placed inside an elastic chest strap to secure it in place. This strap is placed around the chest, with the sensor to the left side, following the ribcage curvature (represented in blue in Figure 2). The PZT sensor characteristics are also described in Table 3, and its transfer function is given by Equation (2).

(2) $$PZT\left(\%\right)=\left(\frac{ADC}{2^{n}}-\frac{1}{2}\right)\times 100\%$$

The electrodermal activity (https://bitalino.com/storage/uploads/media/eda-sensor-datasheet-revb.pdf, accessed on 23 August 2021) (EDA) sensor used in the experiment has two dry AgCl electrodes (https://www.researchgate.net/figure/Resulting-Circuit-parameters-obtained-using-moisture-electrodes-with-artificial-sweat_tbl2_323794069 (first row), accessed on 23 August 2021), where one is placed on the index finger and the other on the ring finger of the left hand (represented in yellow in Figure 2). They are strapped to the finger by adhesive tape. The EDA sensor characteristics are described in Table 3, and the transfer function is given by Equation (3).

(3) $$EDA\left(\mu S\right)=\frac{\frac{ADC}{2^{n}}\times V_{CC}}{0.132}$$

Finally, a photoplethysmography (https://bitalino.com/storage/uploads/media/photoplethysmography-ppg-sensor-datasheet.pdf, accessed on 23 August 2021) (PPG) sensor is also used, with an integrated adjustable velcro fastening strap (placed on the middle finger, represented in green in Figure 2). This sensor uses wavelengths of 520 nm and has a voltage output of 0.3 V to $V_{CC}$.

**Figure 3:**
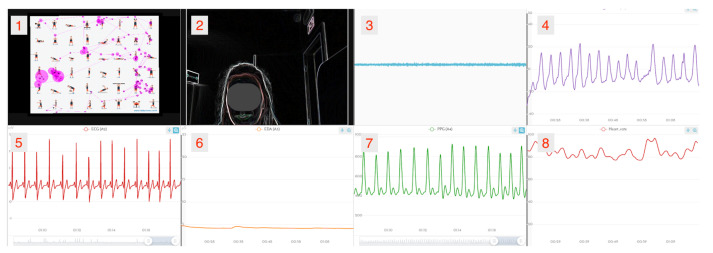
Psychophysiological data obtained during the experiment: (**1**) Eye-tracking, (**2**) Movement and facial expressions (Webcam), (**3**) Sound (Microphone), (**4**) PZT, (**5**) ECG, (**6**) EDA, (**7**) PPG, and (**8**) Heart Rate (HR) derived from the ECG.

The electrodes of every one of the above-mentioned sensors are placed by the participants themselves, through the instructions provided in the form of a video (https://www.youtube.com/watch?v=1WivvUdxbNc, accessed on 23 August 2021) that participants can review autonomously. Figure 3 depicts an example of the results obtained from the acquisition of signals described in this section.

## 3. MobFood Case Study

### 3.1. Overview

For testing purposes, our protocol was applied to a case study project named MobFood (https://mobfood.pt/en/, accessed on 23 August 2021). The focus of this study was to create a breakfast kit composed of three food and beverage products, chosen according to the perceived preferences of a panel of assessors tasting four samples, namely: a cereal bar, cheese, chocolate milk, and an oatmeal cookie. The case study was conducted in the certified sensory analysis laboratory of CATAA (Centro de Apoio Tecnológico Agro-Alimentar) in Castelo Branco, which follows the requirements mentioned in Section 2.2.

### 3.2. Population

The participants in this experiment were recruited by an external company. This was a professional recruitment firm that handled the participant selection, the institutional review board approval, and informed consent. Exclusion conditions from the study included the intake of certain medications (e.g., muscle relaxants and sedatives), as well as specific clinical conditions, such as vision impairment and neurological, cardiac, and dermatological diseases. The results analysed were collected from 83 participants (40 male and 43 female), with ages between 23 and 38 years old (average of 28.80 ± 4.36 years). The participants received monetary compensation for their participation in the study.

There were two training sessions before the actual experiment. These sessions allowed the participants to become familiarised with the protocol, minimising the novelty effect. A shorter protocol was followed in these sessions, involving only two food and beverage samples, one solid (a cookie), and one liquid (a glass of water).

The main experiment was composed of three sessions on different days. Every participant considered in the results participated in the three sessions. The three sessions followed the same protocol and the same type of food samples were used. This approach allows testing repeatability between sessions, in terms of self-reported data and even physiological data.

### 3.3. Methodology

The experiment was performed according to predefined scheduling, for which each participant had a total time slot of 1 h and 30 min allocated (Table 4). In each session, as the participants arrived at their individual booth, they placed the sensors themselves (using an instructional video as guidance), calibrated the eye-tracking software, opened the *BrainAnswer* platform, and started our proposed protocol (Section 2). The completion time of the protocol was between 15 and 62 min, with an average of 29.1 ± 9.0 min. A total of 290 data collection sessions were performed over the course of 3 months.

The main stages to the neuroorganoleptic protocol were: (a) relaxing; (b) preference-ordering tasks with visual stimulus; (c) acceptance assessment questions regarding the samples of each product using the hedonic scales; (d) preference and acceptance questions about the breakfast kit (three of the four products tasted together); and, finally, (e) general questions about food neophobia and socio-demographic characteristics.

The first step involved participants looking at an image meant to relax the volunteers, containing several small figures working out, selected due to the food and beverage neutral content. This first step was important to establish a baseline for the physiological signals and test the eye-tracking calibration.

After this, the protocol proceeded to the preference-ordering tasks with visual stimulus. The main focus of physiological data acquisition in this task was eye-tracking. The visual stimuli in this step were presented to the participants in random order on the screen, and they stayed on the screen for 10 s. Before presenting each set of stimuli images, the participants were requested to stare at a calibration cross in the middle of the screen. The first task of this step was to order the four elements of the kit by preference. The second task was to order a set, including one of the products of the kit and two similar products from competitors, by preference. For instance, the cereal bar of the kit was presented alongside a chocolate cereal bar and an apple cereal bar. This task was repeated four times (one for each product of the kit), and the order of this repetition was random.

Following this came the acceptance assessment questions regarding the samples of each product, using hedonic scales. A product of the kit appeared on the screen, and the assessor then used the switch of the light signal system (connecting the booth to the preparation area), to indicate that he/she was ready to receive the product. The sliding door opened, the assessor used non-verbal communication by giving the paper card with the product, and received the sample from the preparation area. The participant was then asked to assess the visual look of the sample before tasting it, to taste it for 60 s and, following that, to assess the various organoleptic characteristics present in Table 5 using the scales indicated in Section 2 (illustrated in Figure A1). This task was repeated four times, one for each product of the kit, and the order of this repetition was random.

After this came the stage related to the breakfast kit composed of three of the four products studied. In this stage, only visual stimuli were used, and once again they were presented in random order on the screen to the participants, being shown for 10 s. Before each set of stimuli appeared, the participants were requested to stare at a cross in the middle of the screen (neutral content). This step included two tasks, the first one was a preference test in which the assessor had to order two different kits by preference. The only difference between the kits was that one had the oatmeal cookie and the other one had a cereal bar (both included the chocolate milk and the cheese). The second task included answering questions about the chosen kit regarding recommendation, purchase intention, and suitability for breakfast.

Throughout the whole protocol, the assessors were able to leave comments and justify their choices and assessments. Finally, the protocol ended with questions assessing the level of food neophobia and socio-demographic characteristics, such as inquiries about their financial situation and how that affects their expenses on food. These questions are presented in Table A1.

### 3.4. Data Format

All of the data were stored in the *BrainAnswer* platform for more efficient post-processing. In the platform, the data is visually presented in dashboards (Figure 3) and it is possible to select the data sources that the researcher intends to analyse at the same time (e.g., webcam, ECG, etc.). It is also possible to export the BITalino and Gazepoint data in a CSV format, the audio files in a MP3 format, and the webcam recordings in a MP4 format, for further processing. Each recording is labelled with a date/timestamp and the identification code of each participant. Furthermore, this CSV file also includes the indication of the stimulus presentation order in each recording and, in the questions where images with more than one element were presented, the file indicates what was the order of the elements inside the image for that recording. Self-report questionnaire results can also be exported as CSV files.

## 4. Results and Discussion

### 4.1. Qualitative Assessment

Multiple findings have been obtained from the practical application of our neuroorganoleptic protocol in the MobFood case study. Concerning the questionnaires used, improvement points include adding a description of the parameters that are being assessed, such as aroma and texture, rather than just the textual description. Moreover, during the case study, the software presented the scales with the slider in the left end (as it is possible to observe in Figure A1), rather than in the middle of the scale. This could result in biased results and should be amended in future studies.

Furthermore, concerning the psychophysiological signals acquisition, there were some difficulties found during the case study, in both the self-placement of the electrodes by the participants and during the experiment. In what concerns the placement of the different sensors, common problems encountered were (for each sensor):

- ECG: swap of the electrode positions; electrodes placed distantly from the intended position; electrode placement locations not thoroughly clean; high levels of perspiration;
- PZT: strap positioned inside out; strap not properly adjusted or with the cables very stretched; strap on top of the ECG electrodes generating noise due to friction between the two;
- EDA: poorly cleaned fingers; excess perspiration (hyperhidrosis); dry skin (hypohidrosis);
- PPG: sensor placed upside down; sensor too tight; saturated signal due to vasodilation;
- Eye-Tracking: dry eyes (more difficult to calibrate); participants moving after calibration.

Having these caveats identified, it is possible to avoid them in subsequent studies by being alert and preventive about them (as it happened from session to session in the MobFood case study). The instructions for placement could, for instance, include these alerts.

Still regarding the self-placement of the electrodes, and the fact that there were two training sessions and three tasting sessions, some participants considered that they already knew the placement procedure, because they had previously repeated it. This led to skipping or swapping steps, and also being more carefree about aspects such as skin cleansing and accurate electrode placement.

Problems also occurred during the experiment; the main complication found was the temporary or permanent detachment of electrodes as a consequence of movements performed during the experiment. It happened, for example, during the tasting of a drink when the participants raised their arm and tilted their head, in order to drink the entire contents of the glass, and also while eating if the participants lowered the head so that the crumbs would reach the plate. Moreover, when the participant was trying to reach the plate left near the sliding door, motion artifacts were detected in cases where the plate was far from the participant, or when switching the light to indicate they are ready to receive the sample. These movements cause the skin to stretch and shrink, facilitating the movement of the electrodes against the clothing or respiratory belt, leading to the detachment of one or more electrodes.

**Figure 4:**
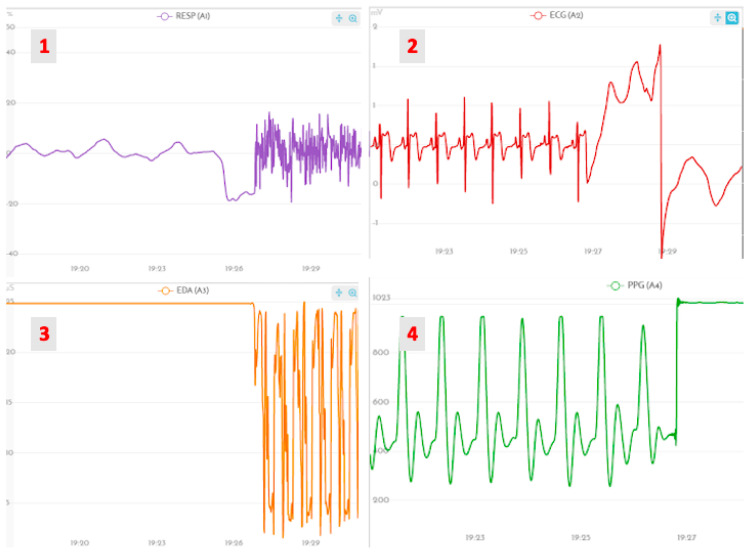
Examples of outlier waveforms obtained for (**1**) PZT, (**2**) ECG, (**3**) EDA, and (**4**) PPG signals.

Figure 4 presents an example of the consequences of the problems described on the physiological data. From this, we conclude that it is important to have the samples and actionable items that the participant needs within easy reach as much as possible. Nevertheless, overall, it is essential that the participants do not get overly conscious about the signal acquisition; otherwise, such concerns might influence the psychophysiological results.

Concerning the psychophysiological data, a final potential improvement point was identified regarding the baseline data acquisition; in the current protocol a visually ’relaxing’ stimuli was used rather than a truly neutral stimuli, such as a blank image. This could be something improved in the following experiments.

### 4.2. Psychophysiological Data Quality and Validity

**Figure A2:**
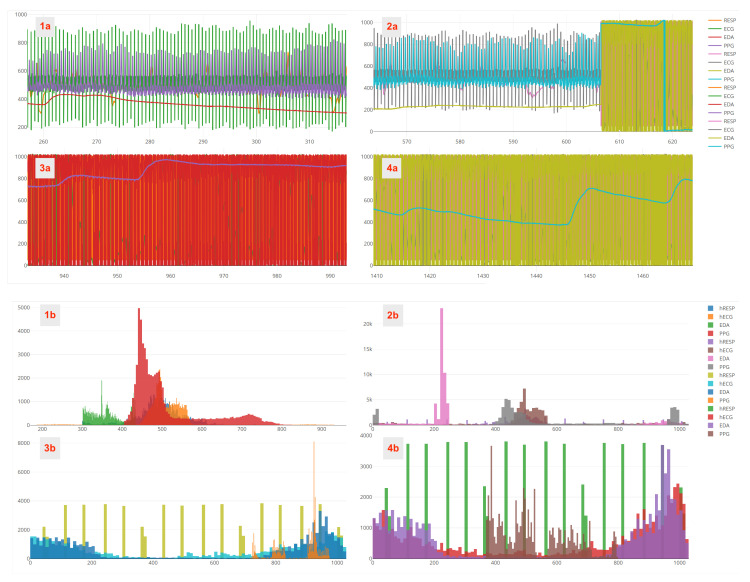
Plots (**top**—**1a**–**4a**) and corresponding histograms (**bottom**—**1b**–**4b**) of the psychophysiological signals acquired in four different moments (**1**–**4**) during one of the sessions of one assessor. It is possible to observe in the plots at the top that in the first moment (**1a**) the signals are valid, in the second moment (**2a**) a failure appears by the end of the acquisition, and in the last moments (**3a**,**4a**) the signals are invalid. From the histogram (bottom), it is possible to observe that, in the first moment (**1b**) when the signals are valid, these are concentrated around a central point; in the second moment (**2b**), the partial signal failure distorts the histogram by reducing the density at central frequencies and a high-frequency component appearing, while in the last moments (**3b**,**4b**) when the signals are invalid, these are dispersed. It was based on this that 3 different clusters (at 20%, 50% and 90% of full scale) were created in order to test the validity of the data.

During the experiment of the case study, a pre-validation algorithm was devised to assess the signals quality. This algorithm, which was run for the data of each assessor, extracted basic characteristics of the biosignals (mean and standard deviation) and calculated the probability of the biosignal belonging to one of three clusters created (centered at 20%, 50% and 90% of full scale). The clusters were created on these points based on the observation of the signals’ histogram (Figure A2). The algorithm flagged a problem, if one of these probabilities were abnormal. For instance, if the ECG signal presented a probability lower than 70% of belonging to the cluster 50% of full scale (corresponding to −0.065 mV), the signal segment was considered noisy and unusable.

This was then validated by checking the time series for such signal segments. The assessor was asked to repeat the session on another day only if there was a problem with the ECG signal; abnormal behaviour in the other biosignals were not considered an exclusion parameter.

Overall, from the 290 sessions, 2 presented a complete loss of signals from one or more sensors, 20 presented severe problems on the signals caused by the detachment of electrodes and 11 presented high levels of noise, caused by excessive movement of the participants. Finally, 15 participants of the initial sample did not gather three valid sessions for the repeatability test, having been excluded from the database.

### 4.3. Self-Report Data

The repeatability of the self-report data between the three sessions was also analysed. Even though there were changes within the same participant across sessions, the overall results of the samples ranking were identical across sessions. This suggests that one session would be sufficient.

Significant differences in the global acceptance were only found for the chocolate milk between the first and last sessions, and for the cookie between the first and the third session (Table 6). A change in the ranking of the visual aspect of the samples at the beginning of the questionnaire did happen between the first and the two last sessions, but that can be explained by the fact that the participants had already tasted the samples previously.

**Figure 5:**
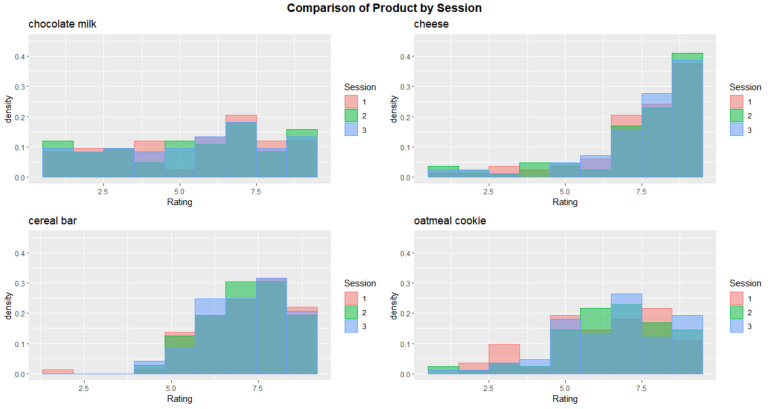
Histogram of the results of the global acceptance for the four products in the three sessions.

**Figure 6:**
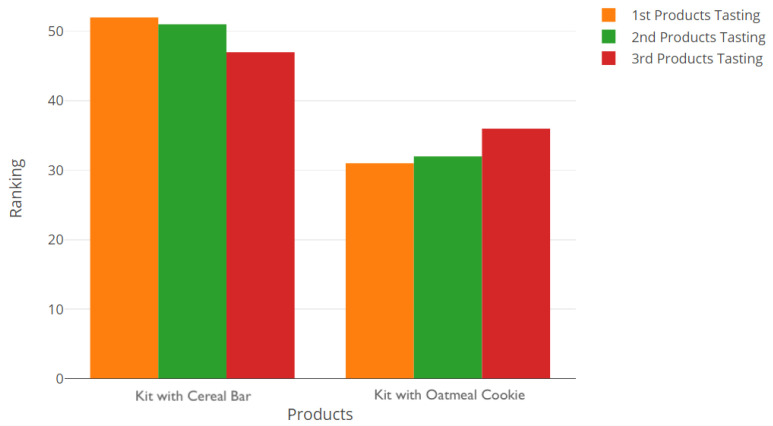
Results of the preference test between a breakfast kit with a cereal bar (**left**) and an oatmeal cookie (**right**) throughout the three sessions.

Comparing the actual results obtained for the global acceptance of the four products (Figure 5), it is possible to see that there was a consistent preference for the cheese, followed by the cereal bar, oatmeal cookie and chocolate milk (in this order). This was further confirmed by the results of the preference test between the cereal bar and oatmeal cookie (Figure 6) since more assessors selected the cereal bar throughout the three sessions. That being said, it was decided that the cereal bar would be part of the breakfast kit (it was beforehand set that the kit would include the cheese and a drink, the chocolate milk; the question was regarding which type of flour-based baked food product would be present).

### 4.4. Psychophysiological Data Analysis

In order to validate the potential of the psychophysiological data dimension of this neuroorganoleptics protocol, the ECG signal was processed and analysed. We focus on this modality, given that heart rate variability (HRV) analysis is frequently present in emotion evaluation studies using psychophysiological data [16,34,35,36].

Given that the ECG may be contaminated by artifacts, the first pre-processing step was outlier detection. For this purpose we used unsupervised learning, more specifically by performing clustering using the DBSCAN algorithm after segmenting the raw ECG samples into heartbeat templates, as described in [37]. The toolbox BioSPPy [38] implements the described method, hence being used in this step. From the results of the clustering, the samples that presented a percentage of the signal belonging to the outlier cluster higher than 5% were eliminated. This threshold value, which ensures the full quality of the samples selected in the relevant time frames, was defined based on the visual assessment of the plots of the signals. Thirty samples of ECG were discarded based on this criteria.

The selected samples were then submitted to a pre-processing step, consisting of a Butterworth bandpass filter of order 3 between 0.5 and 40 Hz. Given that powerline noise persisted, a notch filter in the 50 Hz was also used. After that, the data was normalised and converted to the HRV domain.

After the selection and processing of the ECG samples, feature extraction was performed for relevant sections of the tests (mainly the sections where subjects tasted the food and answered the questions) to check the relation between the results of the self-report questionnaire (more specifically, the classification obtained in the *global acceptance* question) and the extracted features. The focus of this study was on the frequency features HF (High Frequency power) and LF (Low Frequency power), since they have been shown in previous studies to be valid in short-term recordings [34], and because of their relevance in emotional studies [39].

**Figure 7:**
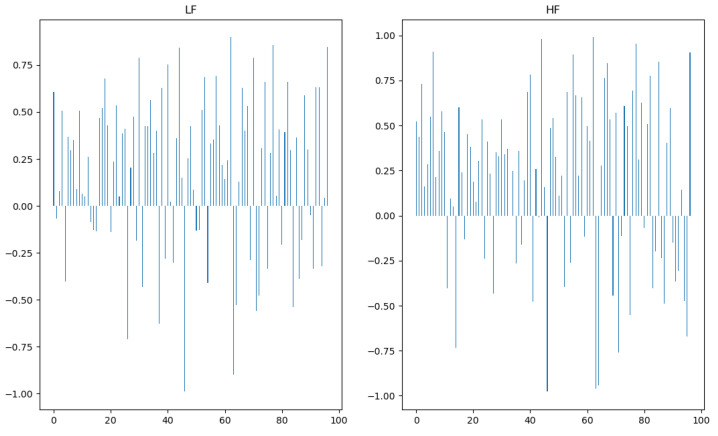
Bar plots of the difference between the normalised values of LF (**left**) and HF (**right**) in the low classification product and the high classification product. The difference in LF and HF was positive for 70% and 69% of this set of 97 experiments, respectively.

Our first step was to investigate if there was a clear difference within each subject between their physiological response to a high and a low acceptance score product. A set of 97 experiments where the subject classified two products with opposed scores in the global acceptance question were selected (the criteria was a minimal difference of five points, e.g., one product got a global acceptance of nine and another got four). For each experiment, the first 2.5 min during which the subject contacted with the product (1 min of tasting +1.5 min of question answering), of the two products that got opposed scores, were analysed to compute the HF and LF features. The most relevant result of this analysis was that for 70% and 69% of the experiments the value of the LF and HF, respectively, was higher for the low acceptance score product (see Figure 7).

**Figure 8:**
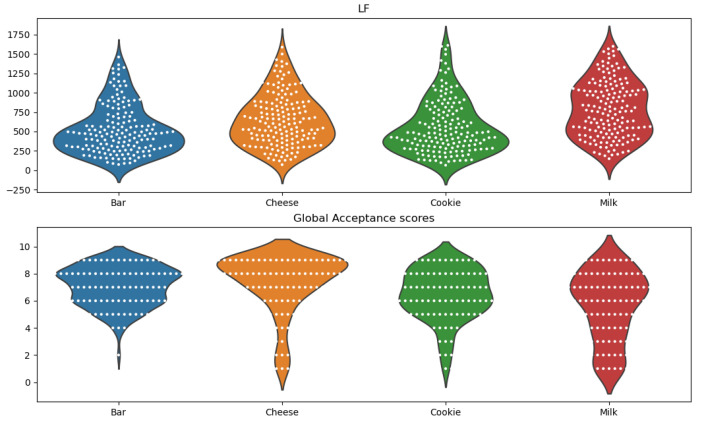
Violin plots representing the distributions of the HRV LF feature values and global acceptance scores for each product across 162 experiments (experiments with outlier values of LF were removed). On **top**: Distribution of LF values obtained in the first 2.5 min of contact with each product. **Bottom**: Distribution of the scores obtained in the global acceptance question.

**Figure A3:**
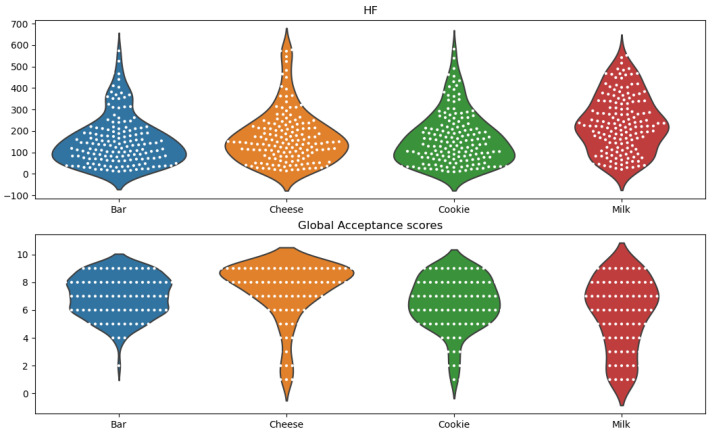
Distribution of the HRV HF feature values and global acceptance score for each product across 156 experiments (experiments with outlier values of HF were removed). On **top**: Distribution of HF values obtained in the first 2.5 min of contact with each product. **Bottom**: Distribution of the classifications obtained in the global acceptance question.

After establishing there were noticeable differences between high/low acceptance score tastings, we focused our attention on checking if there were significant differences between the features obtained from each product, and if these matched the differences between the global acceptance score of the four products. For this purpose, we analyzed the distribution of the HRV power frequency features and global acceptance score for each product through violin plots (LF in Figure 8; HF in the Appendix A Figure A3). The main observation from the plots was that the dispersion of the classifications for the milk product was matched by a dispersion of feature values (for both HF and LF).

Furthermore, for each feature type, a statistic Wilcoxon analysis was conducted to check if there was a significant difference between the feature values obtained for each product and if this matched the differences between the acceptance scores (see Table 7).

Significant differences were found for every product in terms of global acceptance scores. In the features domain, even though most pairs obtained significant differences, it was found that the cookie was harder to distinguish from the bar (neither LF or HF obtained significant difference between the two products) and the cheese (LF obtained a significant difference between the two products, but HF did not). To sum up, the main positive result of this analysis was that through LF it was possible to distinguish the responses to almost every product studied.

## 5. Conclusions and Future Work

In this paper, we described a protocol for organoleptic testing combining self-report questionnaires with video, audio, eye-tracking, and physiological signals (in particular, ECG, EDA, PZT and PPG). In order to prove its applicability and replicability, the protocol was applied to the case study project, MobFood, that aimed to constitute a breakfast kit of three products from the four assessed. From this study, some improvements to the protocol were identified, mostly concerning the acquisition of the psychophysiological signals, either related with the sensor placement or electrode detachment during the experiment. Suggestions and recommendations regarding these factors were presented, allowing future studies to integrate and address them.

Two factors regarding this proposed protocol can be highlighted. First, the methodology for verifying the quality of the signals and the request for repetition when the signals did not present a minimum quality allowed us, in the end, to have a balanced database of physiological signals with 83 participants, 43 female and 40 male, each with three records made on different days. This was a requirement of the study in order to have sufficient data available to study the physiological signals collected from the participants on different days, but subjected to the same stimuli.

Another big step that was achieved with this work was the strategy used to enable the assessors to self-place the sensors. For this, the participants had two training sessions with which they became autonomous regarding the signals acquisition setup. Through the previous analysis of the dataset, we can conclude that it is possible to train assessors, contributing to streamline the protocols.

Regarding the results of the case study, besides the self-report results, it was also possible to obtain some preliminary results regarding the relation between the self-report and psychophysiological data, most specifically the ECG-derived data. Most importantly, it was possible, in most cases, to differentiate the responses between the four products through both the scores of global acceptance and the HRV features considered in this work (LF and HF, although LF obtained better results). A possible connection between higher values of HF and LF and low levels of satisfaction was also suggested by the results, and should be further studied.

Ultimately, by presenting this protocol, recommendations and preliminary results, we seek to raise awareness to the interest (and feasibility) in moving forward from traditional questionnaires in sensory analysis and consumer behavior research. Neuroorganoleptics can contribute to a more holistic approach, in order to accommodate the needs of the increasing competitive food and beverage market.

Future work involves a deeper analysis of the psychophysiological data in order to characterise in more detail possible correlations with the self-report data, across different questions, samples and possibly across the three sessions, based on the initial statistical analysis of the questionnaire results.

## Figures and Tables

**Table 1:** Specifications of *GP3 Eye Tracker* from *Gazepoint*, the eye-tracking software used in the experiment.

Spatial resolution	0.1${}^{{}^{\circ}}$
System latency	<50 ms
Sampling rate	60 Hz
Operating distance	50–80 cm
Calibration	5 or 9 point
Eyewear compatibility	Works with most glasses and contact lenses
Data provided	Timestamp, gaze (x/y coord.), eye position, pupil diameter

**Table 2:** Specifications of *BITalino (r)evolution Plugged BT*, the acquisition unit used in the experiment ${}^{1}$.

Connectivity	Bluetooth
Range	up to 10m
Resolution	up to 10 bit
Sampling rate	1, 10, 100 or 1000 Hz
Size	50 × 65 × 6 mm
Battery	500 mA 3.7 V LiPo (rechargeable)

${}^{1}$https://bitalino.com/storage/uploads/media/revolution-bitalino-plugged-kit-datasheet-2.pdf, accessed on 23 August 2021.

**Table 3:** Specifications of the ECG, EDA, and PZT sensors used in the experiment.

	ECG	EDA	PZT
Bandwidth	0.5–40 Hz	0–2.8 Hz	0.59–0.9 Hz
Range	±1.5 mV	0–25 μS	
Gain	570		
Input impedance	7.5 GOhm		
CMRR	86 dB		

**Table 4:** Factors considered for the experiment scheduling.

Time (in Minutes)	Purpose
10	Maximum lag time for participants to arrive
15	Participants occupy testing booths, place the sensors and verify the signal quality
60	Maximum time for participants to follow the protocol
5	Cleaning and preparation of testing booth for next test

**Table 5:** Questions of the self-report questionnaire and corresponding scales used with the labels of the extremes and middle point indicated.

Question	Left Extreme		Center		Right Extreme	
Assess the visual appearance of the product before tasting it	1	Extremely unpleasant	5	Indifferent	9	Extremely pleasant
Is the product below or above your expectations?		Extremely below		Coincident		Extremely above
Aroma		Extremely unpleasant		Indifferent		Extremely pleasant
Flavor						
Texture in the mouth						
Sweetness						
Evaluation of salt in the product						
Chewability						
The product is satiable		Extremely unsatisfying		Indifferent		Extremely satiable
Global acceptance		I dislike it extremely		I do not like or dislike it		I like it extremely
Preferred consumption temperature		Cold		Room temperature		Hot
From 0 to 10, how likely are you to recommend this product to your friends and family?	0	Never		–	10	With total certainty
Would you buy the product?		Never		I do not know		With total certainty

**Table 6:** Results of the comparison between the results of the global acceptance of the products between sessions using the Kendal method.

Product	Sessions Compared	*p*-Value	rho.tau	
Chocolate milk	1st, 2nd	0.8288	0.0606	uncorrelated
	1st, 3rd	0.112	0.4677	uncorrelated
	2nd, 3rd	0.01	0.7454	correlated
Cheese	1st, 2nd	0.0331	0.5882	correlated
	1st, 3rd	0.0059	0.7537	correlated
	2nd, 3rd	0.0261	0.6088	correlated
Cereal bar	1st, 2nd	0.0011	0.9241	correlated
	1st, 3rd	0.0017	0.8792	correlated
	2nd, 3rd	9e-04	0.9525	correlated
Oatmeal cookie	1st, 2nd	0.0204	0.6288	correlated
	1st, 3rd	0.0747	0.4789	uncorrelated
	2nd, 3rd	0.0148	0.6667	correlated

**Table 7:** *p*-values obtained from the Wilcoxon analysis intended to check significant differences between products concerning features values (HF and LF) and global acceptance scores. The LF analysis was conducted from a set of 162 experiments (set without LF outliers) and the HF analysis from a set of 156 experiments (set without HF outliers). The grey cells indicate pairs where no significant difference was found (*p*-value > 0.01).

	LF Analysis		HF Analysis	
	LF	Global Acceptance	HF	Global Acceptance
Bar vs. Cheese	1.611× 10^−6^	2.978 × 10^−3^	7.547 × 10^−4^	1.735 × 10^−4^
Bar vs. Cookie	3.477 × 10^−1^	1.518 × 10^−4^	9.074 × 10^−2^	3.013 × 10^−3^
Bar vs. Milk	9.944 × 10^−17^	3.467× 10^−8^	2.945 × 10^−19^	2.190 × 10^−6^
Cheese vs. Cookie	3.270 × 10^−4^	2.086 × 10^−5^	3.602 × 10^−2^	6.719 × 10^−6^
Cheese vs Milk	2.385 × 10^−7^	2.007 × 10^−10^	4.515 × 10^−12^	1.953 × 10^−10^
Cookie vs. Milk	2.697 × 10^−12^	1.258 × 10^−3^	9.273 × 10^−14^	3.777 × 10^−3^

## Data Availability

The data are not publicly available due to privacy restrictions, although it may be made privately available upon reasonable request made to the corresponding author.

## Abbreviations

The following abbreviations are used in this manuscript:

ANS	Autonomic Nervous System
ECG	Electrocardiography
EDA	ElectroDermal Activity
HF	High Frequency power
HR	Heart Rate
HRV	Heart Rate Variability
LF	Low Frequency power
PPG	Photoplethysmography
PZT	Piezoelectric Respiration
SNS	Sympathetic Nervous System

## Appendix A

**Table A1:** Food neofobia assessment statements and scale used.

Statement	Left Extreme		Center		Right Extreme	
I constantly try new and different foods	1	Strongly disagree	4	Neither agree nor disagree	7	Strongly agree
I don’t trust new foods						
If I do not know the ingredients of a food, I do not try it						
I like food from different countries						
Ethnic food seems too weird to taste						
At convivial dinners I try new foods						
I am afraid to eat things I have never experienced						
I am selective about the food I eat						
I can eat just about everything						
I like to try new ethnic restaurants						
